# Glucose metabolism reprogramming in gynecologic malignant tumors

**DOI:** 10.7150/jca.91131

**Published:** 2024-03-17

**Authors:** Jianhong Ma, Zhiqiang Yao, Liangjian Ma, Qinyin Zhu, Jiajia Zhang, Ling Li, Chang Liu

**Affiliations:** 1 The First Clinical Medical College of Lanzhou University, Lanzhou, 730000, China.; 2 Department of Obstetrics and Gynecology, the First Hospital of Lanzhou University, Lanzhou, 730000, China.; 3 Department of Child Health, the First Hospital of Lanzhou University, Lanzhou, 730000, China.; 4 Key Laboratory of Gynecological Oncology of Gansu Province, Lanzhou, 730000, China.

**Keywords:** Warburg effect, aerobic glycolysis, cervical cancer, endometrial carcinoma, ovarian cancer, pathological mechanism

## Abstract

The incidence and mortality of gynecological tumors are progressively increasing due to factors such as obesity, viral infection, unhealthy habits, as well as social and economic pressures. Consequently, it has emerged as a significant threat to women's health. Numerous studies have revealed the remarkable metabolic activity of tumor cells in glycolysis and its ability to influence malignant biological behavior through specific mechanisms. Therefore, it is crucial for patients and gynecologists to comprehend the role of glycolytic proteins, regulatory molecules, and signaling pathways in tumorigenesis, progression, and treatment. This article aims to review the correlation between abnormal glucose metabolism and gynecologic tumors including cervical cancer (CC), endometrial carcinoma (EC), and ovarian cancer (OC). The findings from this research will provide valuable scientific insights for early screening, timely diagnosis and treatment interventions while also aiding in the prevention of recurrence among individuals with gynecological tumors.

## Introduction

Cervical cancer (CC), endometrial carcinoma (EC), and ovarian cancer (OC) are the three most prevalent reproductive system tumors in women. Their incidence and mortality rates have been increasing year by year, with a trend towards affecting younger individuals. CC is clearly associated with high-risk human papillomavirus infection (HR-HPV), while the specific causes of EC and OC remain unclear. These diseases pose significant threats to women's life and health. A growing body of evidence supports the close relationship between energy metabolism and the pathogenesis of malignant tumors. In a review article, Yang clarified that pancreatic cancer cells regulate the invasion-metastasis cascade through metabolic transformation from oxidative phosphorylation to glycolysis. This metabolic shift promotes epithelial-mesenchymal transition (EMT), tumor angiogenesis and distant metastasis 1. Feng also reviewed key enzymes, signaling molecules, and carcinogenic pathways involved in aerobic glycolysis, which play crucial roles in regulating proliferation, immune escape, invasion, metastasis and angiogenesis in hepatocellular carcinoma. Ultimately, these processes lead to resistance of liver cancer cells to sorafenib treatment 2. Wu explored the prognostic value of aerobic glycolysis-related factors such as key glycolytic proteins, transcription factors and signaling pathways in breast cancer 3. Kobayashi introduced the therapeutic potential of targeting Warburg effect in gynecological tumors 4. In this review article, we aim to further elucidate both intracellular and extracellular factors contributing to enhanced glycolysis. We will explore the strong association between glycolysis and malignant behavior as well as novel therapeutic approaches targeting glucose metabolism specifically for gynecological tumors.

Catabolic pathways, such as glycolysis, and anabolic pathways, such as mitochondrial oxidative phosphorylation, are interconnected to generate adenosine triphosphate (ATP) for energy production in human cells. In comparison to oxidative phosphorylation, glycolysis provides ATP rapidly but with a lower yield. Key metabolic enzymes associated with glycolysis include hexokinase 2 (HK2), glucose-6-phosphate dehydrogenase (G6PD), lactate dehydrogenase A (LDHA), pyruvate dehydrogenase kinase (PDK) and pyruvate dehydrogenase (PDH). The PDK-PDH pathway plays a crucial role as the central enzyme of the glycolytic network. PDH converts pyruvate into acetyl-CoA, which serves as the link between glycolysis and the tricarboxylic acid cycle (TCA) 5. Decreased expression of PDK alters metabolism from glycolysis towards oxidative phosphorylation. Mitochondria not only produce ATP but also synthesize biological macromolecules including DNA, lipids, proteins and antioxidants 6. Oxidative phosphorylation is regulated by complex biochemical systems involving electron transport and ATP synthesis to maintain cellular homeostasis.

Currently, metabolic reprogramming is a prominent area of research in the field of oncology. There exist significant disparities in metabolism between tumor cells and normal cells. While mitochondrial oxidative phosphorylation serves as the primary energy source for differentiated cells, rapidly proliferating tumor cells rely on glycolysis even under aerobic conditions, a phenomenon known as the Warburg effect 7. The Warburg effect is characterized by enhanced glucose uptake and lactic acid production. Additionally, key players in regulating glycolysis in tumor cells include oncogenes such as c-Myc, tumor suppressor genes TP53 and PTEN, Glucose transporters (GLUTs), oxygen homeostasis regulatory genes like HIF-1α, and glycolytic enzymes including HK, ENO1, LDHA, and PKM2.

The tumor microenvironment provides cancer cells with a specific metabolic milieu rich in nutrients, leading to their metabolic reliance. Cancer cells regulate their metabolism through various mechanisms to meet the energy demands for biosynthesis. For instance, some malignancies primarily depend on glycolysis while others exhibit aerobic oxidation. Coordinated catabolism and anabolism are essential for tumor cells to sustain energy supply and biosynthesis 8. The process of metabolic adaptation is driven by key oncogenic signaling cascades or kinase signals such as HIF-1, microRNAs, adenosine monophospho-activated protein kinase (AMPK) and PI3K/Akt/mTOR. Hypoxia stimulates HIF-1 to induce highly active aerobic glycolysis in the tumor microenvironment, creating an acidic milieu conducive for cancer cell growth. This promotes EMT and leads to more invasive phenotypes like chemoradiotherapy resistance or tumor metastasis 9-11. Such metabolic reprogramming contributes to poor prognosis. Dysregulation of microRNA expression often abnormally activates the glycometabolic enzyme profile either directly or through interactions with other non-coding RNAs, namely GLUT1, LDHA, PKM2 and HK2. This coordination of cell metabolism and proliferative phenotype facilitates malignant transformation 12. Research demonstrates the significance and efficacy of targeted therapy in regulating energy metabolism in malignant tumor cells. AMPK fulfills the rapid growth and proliferation's energy requirements by promoting catabolism while inhibiting anabolism in cancer cells. Additionally, AMPK plays a role in regulating the required energy metabolic reprogramming for stem cell-like differentiation and malignant invasion under metabolic stress 13, 14. In relevant studies, the inhibition of the PI3K/AKT/mTOR signaling pathway has been demonstrated to decrease the expression levels of glycolytic enzymes, leading to apoptosis and restoring chemotherapy sensitivity in cancer cells by reducing ATP energy production, which ultimately improves patient prognosis 15.

Although aerobic glycolysis is a significantly less efficient form of energy production compared to oxidative phosphorylation, increasing glucose intake can compensate for this inefficiency and accelerate glycolysis to generate ample ATP, thereby facilitating cancer cell growth and proliferation. Simultaneously, pyruvate is converted into lactic acid and secreted into the microenvironment, which diminishes local reactive oxygen species (ROS) levels and intracellular oxidative stress, promoting the survival of cancer cells. Moreover, tumor microenvironment acidification activates matrix metalloproteinases (MMPs), enabling extracellular matrix degradation and creating conditions conducive to cancer cell invasion and metastasis. Lastly, heightened glycolytic activity also supplies macromolecular substances for cellular biosynthesis. These functions collectively confer growth advantages upon cancer cells and targeting aerobic glycolysis could potentially yield a promising anticancer therapeutic strategy. Given that different tumors possess distinct metabolic characteristics while cancer cells often exhibit a mixed phenotype involving both aerobic glycolysis and oxidative phosphorylation, it becomes crucial to identify the metabolic traits for each gynecological tumor in order to develop personalized treatment regimens. Therefore, gynecologic oncologists must thoroughly consider the impact of metabolic heterogeneity prior to initiating tumor treatment by devising accurate non-invasive methods for early detection of metabolic alterations that facilitate optimal diagnosis and treatment decision-making.

## Glucose metabolism in CC

According to the 2018 Cancer Statistics 16, there were approximately 569,847 new cases and 311,365 deaths worldwide due to CC. This corresponds to an incidence rate of 6.6% and a mortality rate of 7.5%, ranking it fourth globally. Developing countries account for over 80% of these cases, making CC the second leading cause of cancer-related death among women in those nations. The prognosis for patients with advanced stage disease is poor, with a five-year survival rate of only around 15% 17. High-risk factors associated with CC include human papillomavirus (HPV) infection, malnutrition and multiple sexual partners. Despite significant advancements in treatment, the incidence and prevalence of CC continue to rise each year. Therefore, there is an urgent need to elucidate its pathogenesis and improve treatment outcomes.

### Key enzymes of aerobic glycolysis in CC

#### Hexokinase 2 (HK2)

Hexokinase 2 (HK2), the initial rate-limiting enzyme in glucose metabolism, catalyzes the conversion of glucose to glucose-6-phosphate (G-6-P) and exhibits significantly increased expression in cervical cancer tissues. Firstly, HK2 interacts with various non-coding RNAs to regulate CC's glucose metabolism. Yang confirmed that miR-148a inhibits endogenous HK2 expression, thereby impeding stem cell characteristics and cisplatin chemotherapy resistance in CC cells by interfering with the expression of stem cell-related genes (such as SOX2, OCT4) and multidrug resistance gene MDR1. Consequently, targeting sphere formation ability in CC cells could potentially eliminate a subset of cancer stem cells (CSCs) and restore cisplatin sensitivity in patients 18. Similarly, Liu discovered that miR-9-5p suppresses HK2 expression, deactivates the Akt/mTOR signaling pathway, enhances p53 activity, subsequently restraining proliferation and migration while promoting apoptosis in SiHa cell line 17. Secondly, Fan demonstrated that long non-coding RNA (lncRNA) UCA1 may target HK2 to augment glycolysis processes leading to local radiotherapy resistance in CC. The inhibition of HK2 using 2-DG can restore radiosensitivity by blocking glycolysis, it would provide a novel target for enhancing radiotherapy efficacy 19.

Several studies have also utilized omics technology to screen for HK2-related oncogenes and pathways. For instance, Chen conducted transcriptome sequencing analysis on HeLa cells and discovered that overexpression of HK2 activated the Akt1 signaling pathway, resulting in increased expression of FN1, MMP2 and MMP9. This activation enhanced cell viability and distant metastasis ability. Conversely, phosphorylation of Akt1 signaling also influenced the expression level of HK2, indicating a feedback loop between HK2 and Akt1 signaling that synergistically regulates abnormal glucose metabolism and impacts malignant progression in CC 20. Cui also demonstrated that HK2 activates ERK1/2 signaling through the Raf/MEK cascade, subsequently inducing cyclin A1 expression. This induction promotes cell proliferation and tumor formation by accelerating cell cycle progression 21. These findings suggest that targeting HK2 could be a potential therapeutic strategy as well as a prognostic indicator for CC.

#### Lactic dehydrogenase A (LDHA)

The expression of lactic dehydrogenase A (LDHA) is upregulated in numerous tumors. A cohort study was conducted by a group, which included 150 CC patients with a median survival time of 38 months who were to receive first-line radiotherapy. It was discovered that patients with higher levels of CRP and LDH before radiotherapy had worse overall survival (OS) and progression-free survival (PFS), confirming the poor predictive value of serum CRP and LDH in advanced patients 22. In terms of mechanism, Shao reported that knockdown of long non-coding RNA NEAT1 suppressed LDHA expression and glycolytic rate by upregulating miR-34a but enhanced the sensitivity of cancer cells to 5-Fu chemotherapy, suggesting that targeting LDHA could be a promising therapeutic strategy for drug-resistant CC 23. Multiple studies have demonstrated that CDKN2A downregulates LDHA expression, inhibits LDHA-mediated Akt/mTOR and JNK signaling pathways, impairs energy metabolism, further inhibits the Warburg effect, induces G2/M cell cycle arrest and activates mitochondrial apoptosis pathway in HeLa and SiHa cell lines. Consequently, there was inhibited cell viability, proliferation, colony formation indicating the oncogenic role of LDHA as well as its clinical value as a therapeutic target 24, 25. Additionally, Liu demonstrated that HR-HPV infection relies on ROS-induced nuclear translocation and dimerization of LDHA to acquire atypical enzyme activity. The production of antioxidant metabolite α-hydroxybutyric acid promotes DOT1L interaction with LDHA triggering histone methylation. This not only protects cancer cells from oxidative stress damage but also activates Wnt signaling pathway regulating CC progression 26. Therefore these studies provide valuable insights into anti-cancer therapy targeting LDHA while also recommending exploration into the carcinogenic role played by other key enzymes involved in the Warburg effect.

#### Pyruvate dehydrogenase kinase (PDK)

The pyruvate dehydrogenase kinase (PDK), a crucial checkpoint regulating the transition from aerobic glycolysis to the tricarboxylic acid cycle (TCA cycle), phosphorylates the ser293 site of the pyruvate dehydrogenase complex, thereby inhibiting its activity. This metabolic shift redirects energy metabolism from oxidative phosphorylation towards aerobic glycolysis 27. Various miRNAs target PDK expression to modulate glycolysis and impact apoptosis and autophagy protein levels, as well as the metabolic characteristics of Adriamycin-resistant cells. Consequently, these regulatory mechanisms further influence proliferation, migration, invasion, autophagy activity and chemotherapy resistance in CC tumor cells 28-31. This research elucidates the correlation between glycolysis and malignant tumor behavior while highlighting PDK subtypes—particularly PDK1 and PDK4—as potential therapeutic targets in CC.

#### Pyruvate kinase M2 (PKM2)

Pyruvate kinase M2 (PKM2) is a crucial enzyme that regulates glycolytic metabolism and catalyzes the conversion of phosphoenolpyruvate and ADP to pyruvate and ATP, thereby acting as a rate-limiting factor. The expression level of PKM2 is elevated in cervical cancer tissues and cell lines, which is closely associated with drug resistance. Liu et al. reported that knockdown of PKM2 leads to increased phosphorylation of cell cycle checkpoint proteins including ATM, Chk1, γ-H2AX, accompanied by p53 activation. This process results in decreased expression of CSC markers and increased levels of apoptotic proteins, ultimately enhancing radiosensitivity through induction of apoptosis, G2/M cell cycle arrest and prevention of DNA damage repair 32. These findings suggest that targeting PKM2 may represent a promising strategy to improve the efficacy of radiotherapy in cervical cancer. Additionally, Wang et al. discovered that overexpression of Cdc25A dephosphorylates nuclear PKM2, conferring protection against autophagy-mediated ferroptosis and further promoting sorafenib resistance. This study reveals a novel mechanism for overcoming drug-resistant cervical cancer 33.

STIP1 downregulates PKM2 expression and utilizes glycolytic metabolism as an intermediate bridge to indirectly inhibit the proliferation, migration, and invasion of cervical cancer cells 34. Zhang discovered that PCBs stimulate HeLa cells to produce ROS through NADPH oxidase, which acts as an upstream signal to induce PKM2 expression and nuclear translocation. Nuclear PKM2 functions as a protein kinase that promotes the transcription of glycolytic genes such as GLUT1, ALDH and PDK, providing energy for cell survival 35. Liu confirmed that inhibiting PKM2 also prevents EMT process by Wnt/β-catenin signaling pathway and thus slows down the progression of cervical cancer 36. This study provides a new treatment target for the tumor.

#### Other glycolytic enzymes

Glycolytic enzymes, such as aldolase A (ALDOA) and enolase 1 (ENO1), are dysregulated in CC. Among these, ALDOA induces the expression of hypoxia-inducible factor-1α (HIF-1α), leading to typical EMT-like morphological changes in cancer cells, including a transition from cuboid to spindle shape and loss of microvilli. Concurrently, there is also a decrease in the expression of adhesion proteins and tight junction proteins, further promoting a malignant invasive phenotype in cancer cells 9.

### Glucose transporter (GLUT) in CC

It is crucial for cancer cells to upregulate the expression of specific glucose transporter (GLUT) in order to enhance energy metabolism and increase the demand for glucose. This mechanism plays a key role in maintaining a high glycolysis rate by promoting glucose uptake. The high expression of GLUT1 is closely associated with age≥50 years, squamous cell carcinoma, advanced stage, low grade and pelvic lymph node metastasis. Consequently, patients with these characteristics exhibit poor PFS and OS 37. Wang utilized TCGA database analysis to demonstrate that elevated GLUT1 expression interacts with DNAJC8 to disrupt glucose metabolism in CC, resulting in reduced infiltration of immune cells such as CD8+ T cells, B cells and Th1 cells within the tumor microenvironment. This immune escape phenomenon renders HPV16+ patients less responsive to treatment 37, 38. These findings suggest that targeting GLUT1 could be a potential therapeutic strategy for CC by reversing GLUT1-mediated immune escape and inhibiting tumor progression.

### Regulatory molecules of aerobic glycolysis in CC

#### P53

Several studies have confirmed that p53, a tumor suppressor gene, modulates the energy metabolism of CC cells and interacts with Fra-1, a member of the Fos family. On the one hand, Fra-1 mediates the p53 signaling pathway through STAT1 to increase intracellular Ca2+ concentration while reducing ROS levels, mitochondrial membrane potential and the expression of multiple glycolytic enzymes. This restoration of mitochondrial function inhibits the Warburg effect and fatty acid metabolism in HeLa cells ultimately leading to suppressed cancer cell proliferation and promotion of senescence and apoptosis 39. On the other hand, Fra-1 inhibits aerobic oxidation in cancer cells through the MDM2/p53 pathway while promoting glycolysis and pentose phosphate pathways to induce malignant progression 40. These studies provide a theoretical basis for energy metabolism therapy in CC. Wang demonstrated that SMYD2 can methylate p53 at site K370 thereby inhibiting its transcriptional activity which promotes CC occurrence dependent on aerobic glycolysis 41. Hernández indicated that mutant p53^R248Q^ significantly reduces adhesion protein expression such as E-cadherin and β-catenin but upregulates CDKN1A, NF-κB, c-Myc and HIF-1α levels resulting in increased glycolytic flux and decreased oxidative phosphorylation 42. The experiment observed higher cell viability and shorter proliferation time suggesting value for developing novel anticancer drugs targeting hyperglycolytic tumor cells.

#### Hypoxia inducible factor-1(HIF-1α)

The rapid proliferation of tumor cells often leads to local hypoxia, but they can obtain sufficient energy to maintain physiological activities in the hypoxic environment. This ability is closely associated with the activation of hypoxia-related genes, such as HIF-1α. Insufficient glucose and oxygen supply, p53 mutation or G6PC overexpression can induce an increase in HIF-1α levels within the tumor microenvironment 10, 42, 43. HIF-1α then induces the expression of GLUT1, HK and ALDOA to accelerate glycolysis rate and subsequently promote cancer cell invasion and migration through mechanisms involving EMT and chemoradiotherapy resistance that ultimately lead to poor survival outcomes 9, 10. Hypoxia also enhances the expression of lncRNA OIP5-AS1 in CC cells. Li confirmed that OIP5-AS1 inhibits miR-124-5p while promoting isocitrate dehydrogenase expression 44. Furthermore, HIF-1α promotes the Warburg effect in cancer cells and augments autophagy activity mediated by BCL2 and BNIP3. These effects contribute to pro-tumor processes such as cell proliferation and migration 44, 45.

#### MicroRNAs (miRNAs)

MicroRNAs (Table 1), such as miR-148a, miR-9-5p and miR-34a, play a crucial role in the occurrence and development of CC by negatively regulating the expression of glycolytic enzymes. These microRNAs directly bind to the promoter region of HK2 and inhibit its expression 17, 18, 23. MiR-126-3p, miR-103a-3p and miR-16-5p target PDK to suppress its transcriptional and translational activity 28, 30, 31. Additionally, miR-34a inhibits PKM2 expression 23. By modulating key enzyme activities involved in glucose uptake and lactate production regulation, these microRNAs exert negative control over malignant behavior of CC cells while restoring sensitivity to chemotherapy drugs. Moreover, MiR-155-5p regulates the PDK1/mTOR pathway to inhibit LC3 but promote P62 protein expression in cancer cells, thus it further influences cellular autophagy activity 29. LncTDRG1 targets miR2145-p to enhance SEMA4C expression which is involved in hypoxia-induced glycolysis regulation, which subsequently affects invasion process of CC cells 46. These findings highlight a complex ceRNA carcinogenic regulatory network.

#### c-Myc

C-Myc, a key regulator of aerobic glycolysis, has a high level in CC tissues and positively correlates with tumor stage. The oncoproteins E6 and E7 of HR-HPV induce m6A methylation modification of c-Myc mRNA by activating IGF2BP2, thereby promoting aerobic glycolysis, proliferation and metastasis in CC. This establishes a significant association between aerobic glycolysis and cancer progression 47. Blocking HPV E6/E7 related metabolic pathways could be a potential strategy for CC patients. Dai utilized TCGA data and clinical samples to demonstrate that HOXC13 activates Myc protein through β-catenin signaling pathway and regulates the aerobic glycolysis pathway by binding to the promoter regions of HK2, PKM2, PDK1, GLUT1, and LDHA in CC cells 48. It exerts carcinogenic effects by inducing cell cycle progression as well as promoting proliferation, migration, invasion and cisplatin resistance (Supplementary Figure 1).

### Signal pathway of aerobic glycolysis in CC

**Figure 1:**
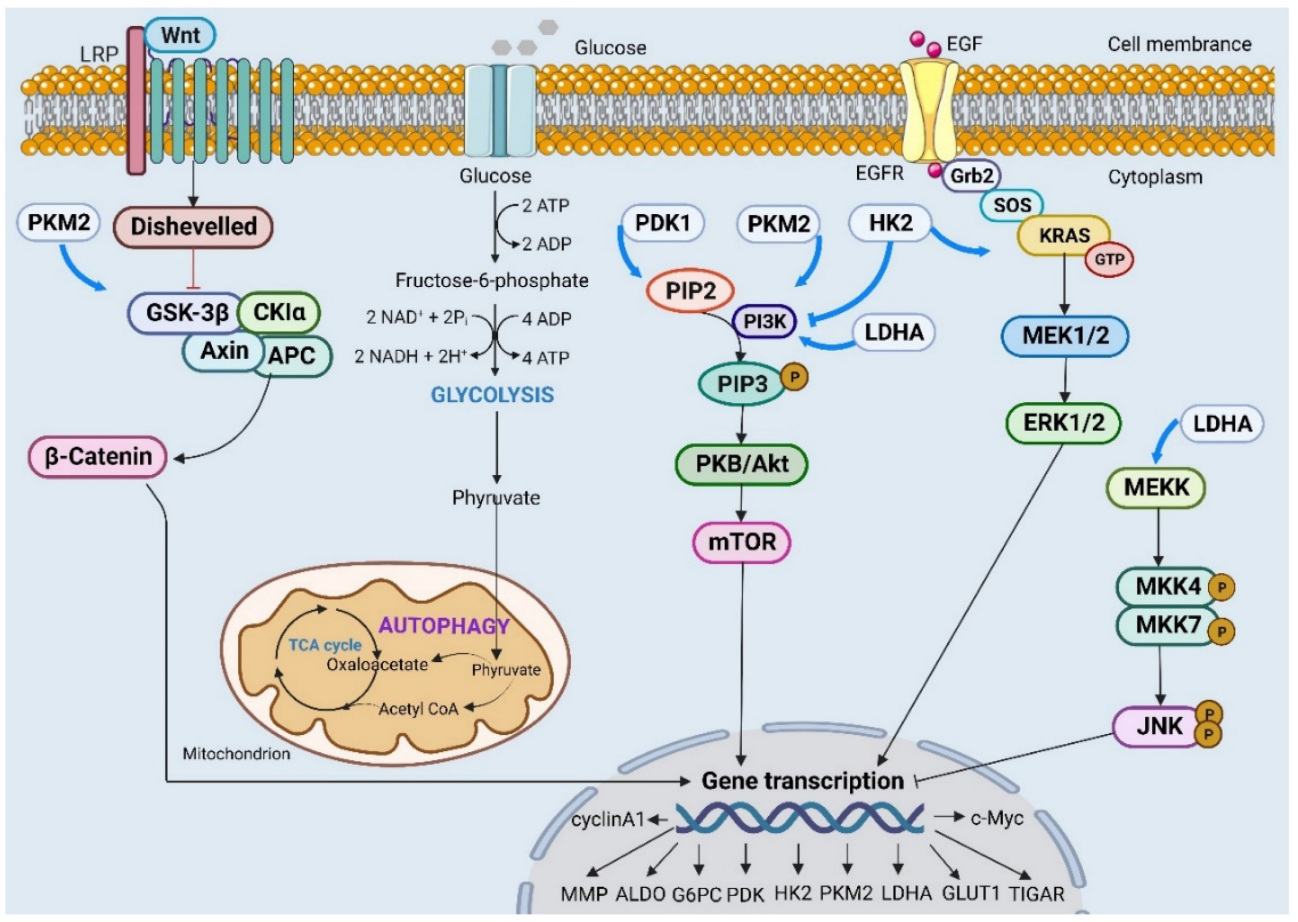
Different signaling pathways affect the expression of key regulatory genes through different mechanisms and then participate in malignant behaviors such as proliferation, invasion and migration in cervical cancer cells.

The Akt signaling pathway is considered the most critical carcinogenic pathway in CC, as it plays a crucial role in regulating the expression of glycolytic proteins, including HK2, LDHA, PDK1, and G6PC. These proteins control malignant behaviors such as unlimited growth, distant metastasis and angiogenesis in CC 17, 24, 28, 43. CNPY2 and TKTL1 activate the Akt pathway to facilitate glycolysis in CC cells. This activation leads to increased glucose consumption, lactate secretion and ATP production. Inactivation of the Akt pathway by targeting these molecules could suppress the malignant process 49, 50. HR-HPV infection negatively regulates miR-155-5p expression while activating Akt/mTOR signaling to promote glycolytic flow. This activation also contributes to chemotherapy resistance with drugs like 5-Fu and enhances cell autophagy activity 29, 51 (Figure 1).

### Human papillomavirus (HPV) infection of aerobic glycolysis in CC

Approximately 95% of cervical cancer cases are attributed to persistent infection with high-risk human papillomavirus (HR-HPV), and its viral oncogene plays a pivotal role in the process of cervical carcinogenesis. The oncoproteins HPV 16/18 E6/E7 directly stimulate the expression of HK2 and PKM2, activate HIF-1α, or influence the binding between IGF2BP2 and Myc to indirectly regulate glycolytic protein activity. This leads to an increased reliance on glycolysis metabolism by elevating extracellular acidification rate while reducing oxygen consumption 37, 47, 52, 53. Consequently, these oncoproteins govern CC pathogenesis and radiosensitivity. Moreover, HPV16/18 E7 methylates H3K79 to enhance antioxidant gene expression and activates Wnt signaling pathway that promotes cell survival and proliferation under oxidative stress conditions 26. Therefore, targeting HR-HPV infection as a means to combat glycolytic flux represents an effective strategy for CC treatment.

### Therapeutic value of aerobic glycolysis in CC

The drug metformin, commonly used for diabetic patients, has garnered significant attention from the scientific community due to its anticancer properties. Metformin not only directly regulates the activity and subcellular localization of HK2, leading to the dissociation of HK2 from mitochondria and subsequent destruction of glycolysis while enhancing oxidative phosphorylation, but also downregulates pathway proteins such as mTOR, MIB and MGMT. This inhibition of tumor growth and induction of apoptosis 52 is further complemented by metformin's ability to enhance the sensitivity of HPV+ cancer cells to radiotherapy. By targeting glycolytic proteins within the metabolic network, metformin broadens the concept of anti-cancer treatment. Additionally, it activates AMPK signaling to disrupt energy homeostasis in cancer cells by downregulating intracellular concentrations of HIF1α and c-Myc. This redirection of carbon flux towards TCA cycle increases ROS production inducing energy stress that can impair biosynthesis and antioxidant protection mechanisms in cancer cells. Combining cisplatin with metformin could potentially enhance drug activity and accelerate apoptosis in cancer cells 54. These studies highlight the feasibility of developing personalized treatment systems based on specific antioxidant systems. Furthermore, berberine reduces levels of p-PI3K and HIF-1α while overcoming radioresistance caused by low glucose and hypoxia. It provides an effective approach for regulating glucose metabolism to enhance radiotherapy efficacy 10.

In summary, the glycolytic enzymes HK2, ALDH, PKM2 and GLUT1 exert their influence on the malignant invasion behavior and therapeutic efficacy of radiotherapy and chemotherapy in CC through distinct mechanisms. It has been confirmed that they hold potential as diagnostic and treatment markers for CC. Key signaling pathways in CC involve common signaling molecules such as p53, HIF-1α, Myc and miRNAs which play a crucial role in regulating glucose metabolism. Additionally, HR-HPV infection may impact cellular glucose metabolism processes and contribute to the pathogenesis of CC. Drugs targeting glucose metabolism regulation like metformin and berberine are anticipated to enhance patient survival rates while delaying disease progression.

## Glucose metabolism in EC

The incidence of endometrial carcinoma (EC), the most prevalent gynecological malignancy in developed countries, is steadily increasing and affecting younger populations due to various factors including obesity, diabetes and hyperlipidemia. In 2020 alone, there were approximately 65,620 new cases and 12,590 deaths reported 15. Some patients are diagnosed with local or distant metastasis at the time of initial diagnosis. Therefore, it is crucial to explore novel and effective screening techniques as well as treatment options for EC patients who are unsuitable for surgery but desire to preserve their reproductive function or may experience late recurrence.

### Key enzymes of aerobic glycolysis in EC

#### Hexokinase 2 (HK2)

The long non-coding RNA DLEU2 functions as an upstream activator that not only competitively binds to miR-455, inducing HK2 expression, but also interacts with EZH2 to suppress miR-181a, further stimulating HK2 expression in EC tissues and cells. Additionally, SNHG9 directly upregulates HK2 expression, and the overexpression of HK2 activates focal adhesion kinase (FAK) and its downstream ERK1/2 signaling pathway to enhance aerobic glycolysis in EC cells. Consequently, this promotes cancer cell proliferation and EMT-induced invasive growth. These findings contribute to a better understanding of the intricate glucose metabolism in EC cells and provide valuable mechanistic insights for tumor treatment 55, 56.

#### Pyruvate kinase M (PKM)

High expression of PKM2 is significantly associated with a poor prognosis in endometrial cancer. Hosseini confirmed that the presence of PKM1 can predict the progression from endometrial atypical hyperplasia to invasive carcinoma, and the combined detection of PKM1 and PKM2 can serve as biomarkers for distinguishing normal endometrium, precancerous lesions and EC 57. In mechanistic studies, estradiol up-regulates PKM expression and facilitates the formation of PKM2 through c-Myc/hnRNP splicing. This process disrupts its tetramer structure and promotes nuclear translocation. Nuclear localization of PKM2 induces the Warburg effect, providing bioenergy, anabolic precursors and redox equivalents for cancer cell growth and proliferation 58.

#### Aldehyde dehydrogenase (ALDH)

The level of ALDH is an independent risk factor for both PFS and OS in EC patients. Mori discovered that high expression of ALDH is enriched in subsets of CSCs within cell lines, leading to increased invasiveness and self-renewal ability during tumor progression 59. Mechanistic studies have demonstrated that elevated levels of ALDH upregulate GLUT1, promoting glucose uptake which is a key pathway for maintaining CSCs and paclitaxel resistance. This research suggests that the synergistic use of paclitaxel and ALDH inhibitors may be a novel strategy to combat CSC components, potentially improving therapeutic efficacy while prolonging survival cycles in EC patients 59.

#### Other glycolytic enzymes

PFK158, a specific inhibitor of Fructose 2,6-Bisphosphatase 3 (PFKFB3), effectively suppresses glycolysis rate to impede cancer cell proliferation and enhances the sensitivity of cancer cells towards carboplatin and cisplatin. Its mechanism involves elevating the level of γ-H2AX in cancer cells to induce DNA damage, down-regulating RAD51 to hinder DNA repair processes, thereby restoring chemotherapy sensitivity. The combined action of PFK158 and platinum agents synergistically inhibits Akt/mTOR signaling pathway leading to cell death mediated by apoptosis and autophagy. It is speculated that the combination therapy involving metabolic enzyme inhibitors and platinum drugs may serve as a novel therapeutic strategy for improving efficacy and prognosis in advanced or recurrent patients 15. Phosphoglucose isomerase (PGI) is an enzyme responsible for catalyzing the reversible conversion between glucose-6-phosphate and fructose-6-phosphate, while also functioning as a cytokine known as autocrine motility factor (AMF). It possesses dual roles as both a glucose-catalyzing enzyme and cytokine. Li integrated exon sequencing data, RNA sequencing data along with clinical information from 587 EC patients, revealing that levels of AMF/PGI along with its membrane surface receptor AMFR/gp78 are elevated in POLE mutant EC patients. This elevation correlates with downstream signal transduction activation regulating glycolysis and gluconeogenesis in cancer cells, ultimately normalizing glucose metabolism to improve patient clinical outcomes 60.

### Glucose transporter (GLUT) in EC

Studies have demonstrated a close association between GLUT1 expression and prognostic indicators such as advanced stage, low grade, lymphatic metastasis and muscular infiltration in EC patients. Gu's research revealed that high glucose levels induce the expression of GLUT4 through ERα and ERβ. This upregulation of GLUT4 not only enhances VEGF and its receptor levels but also promotes the transcription of EMT-related genes (TWIST, SNAIL, CTNNB1), initiating the EMT process and invasive growth by stimulating tumor angiogenesis. Additionally, it facilitates increased glucose uptake to provide ample energy supply for tumors, ultimately accelerating EC progression 61. Caruana discovered that TNFα activates the NF-κB signaling pathway specifically to regulate obesity-related GLUT6 expression, thereby promoting glycolysis-associated carcinogenic signals. Their study elucidated distinct mechanisms targeting different subtypes of GLUTs to regulate glucose metabolism in EC patients, offering new insights for diagnosis and treatment 62. Zeng found that LEFTY2 increases Sodium/Glucose Cotransporter 1 (SGLT1) abundance and glycogen synthetase activity. When extracellular glucose concentration is lower than intracellular concentration, SGLT1 utilizes its electrochemical gradient to facilitate transmembrane transport of glucose coupled with Na+ influx into the plasma membrane 63. However, LEFTY2 inhibits Na+/H+ exchanger activity leading to decreased glycolytic flux while simultaneously stimulating glycogen synthesis as an energy reserve for cancer cells 63.

### HIF-1α of aerobic glycolysis in EC

The expression of HIF-1α is significantly elevated in EC tissues compared to adjacent normal tissues, serving as a prognostic indicator for poor outcomes in EC. Higher levels of HIF-1α are associated with an increased risk of recurrence. Mechanistically, Gong discovered that AGR2 interacts with MUC1 to induce the expression of HIF-1α, which subsequently regulates key proteins including ALDH, HK2, ENO1 and GLUT1. This induction leads to a shift from oxidative phosphorylation to glycolysis, promoting tumor angiogenesis and aggressive phenotype development. Consequently, these factors contribute to unfavorable overall survival rates in patients. These findings suggest that HIF-1α may serve as a valuable tool for pathological screening and prognosis evaluation 64 (Supplementary Figure 2).

### Signal pathway of aerobic glycolysis in EC

ABHD5 activates the Akt/mTOR signaling pathway in EC and induces the expression of key glycolytic proteins and EMT regulatory molecules, thereby exerting a carcinogenic role through the regulation of glycolytic activity and EMT process 7. Estradiol also phosphorylates PKM2 by activating the PI3K/Akt/mTOR pathway to promote the Warburg effect. However, combined treatment with PFK158 and platinum-based chemotherapy synergistically inhibits the Akt/mTOR cascade, thus counteracting the malignant potential in EC cells 15, 58. Studies have demonstrated that the MAPK pathway is involved in regulating carcinogenic activity in EC. Han revealed that PIM2 phosphorylates AMPKα1 leading to a decrease in its activity, subsequently promoting aerobic glycolysis and tumor growth. This suggests that combining a PIM2 inhibitor with an AMPKα1 activator may represent a potential strategy for improving prognosis among EC patients 65 (Supplementary Figure 2).

### Therapeutic value of aerobic glycolysis in EC

Diabetic patients have a twofold increased risk of EC and a 41% higher mortality rate compared to non-diabetic patients 66. This finding suggests that controlling blood glucose levels or interfering with related molecular signals could provide novel treatment strategies for diabetic EC patients. Metformin functions as a mitochondrial complex I inhibitor, impeding electron transport in the respiratory chain. This leads to an increase in mitochondrial mass while reducing their activity. Consequently, it promotes the shift of glucose metabolism from oxidative phosphorylation to glycolysis and inhibits the pro-proliferation pathway, thereby exerting anticancer effects 67. Furthermore, mass spectrometry proteomics analysis revealed a significant upregulation of PDK1 induced by high glucose in the Ishikawa cancer cell line. This further enhances the malignant biological behavior of the cell line through activation of the AKT/GSK3β/β-catenin signaling pathway. Combining a PDK1 inhibitor with metformin results in a 2.5-fold inhibition of proliferation rate and a 5.2-fold increase in apoptosis rate compared to metformin alone in Ishikawa cells with high glucose levels. Additionally, this combination significantly reduces tumor growth in diabetic mice. The mechanism underlying this dual inhibition involves targeting both glycolysis and oxidative phosphorylation, providing a novel therapeutic strategy for patients with diabetic endometrial cancer 66.

In accordance with current research, numerous studies have also substantiated the impact of key glycolytic proteins and transcription molecules on the occurrence and progression of EC through diverse mechanisms and signaling pathways. It is widely acknowledged that EC is a hormone-dependent tumor, wherein hormone-related IGF and ER play crucial roles in pathogenic regulation via glycolysis. Furthermore, genes associated with glycolysis also regulate the immune evasion process of tumors. Apart from aerobic glycolysis, gluconeogenesis is also implicated in the pathogenesis of EC. Metformin not only mitigates the hyperglycemic microenvironment to reduce EC incidence but also enhances survival rates among patients with diabetes-associated EC while delaying disease progression.

## Glucose metabolism in OC

Ovarian cancer is the most lethal gynecological malignancy, commonly referred to as the "silent killer". The incidence and mortality rates have been steadily increasing, with nearly 300,000 new cases and 185,000 deaths reported worldwide in 2018 16. Due to the absence of distinct symptoms and reliable early-stage screening methods, approximately 80% of patients are diagnosed at an advanced stage with extensive abdominal metastasis. Despite aggressive cytoreductive surgery performed by gynecologists followed by well-regulated chemotherapy, a majority of patients experience relapse and develop resistance to chemotherapy. This leads to a discouraging 5-year survival rate ranging from only 15-30%, with a median survival time of merely one year after drug resistance emerges 68,69. Therefore, it is imperative to elucidate the molecular mechanisms underlying OC occurrence and progression, identify biomarkers for early detection through screening methods and devise novel treatment strategies aimed at improving patient prognosis.

### Key enzymes in aerobic glycolysis in OC

#### HK2

The overexpression of HK2 in ovarian cancer leads to resistance against chemotherapy. Elucidating the role of HK2 in tumorigenesis and chemotherapy resistance would facilitate the identification of novel anti-cancer therapeutic strategies.

Mechanistically, Yu demonstrated that mutations in the UBA domain of the autophagy receptor p62 alter the overall abundance and mitochondrial localization of HK2 in OC cells. Subsequently, phosphorylated HK2 activates parkin and ERK1/2, thereby enhancing mitophagy activity, ultimately resulting in cisplatin resistance and cancer cell survival 13, 83. These findings support the notion that HK2 acts as a mediator promoting chemoresistance by augmenting drug-induced autophagy activity. Li also discovered that the SBD domain of HSP70 induces mitochondrial expression of HK2 to increase mitochondrial membrane potential, consequently promoting cisplatin resistance and apoptosis resistance in cancer cells 84. His research provides robust theoretical evidence for considering HK2 as a target for overcoming drug resistance while offering a scientific basis for developing HK2 inhibitors.

The tumor suppressor gene p53 modulates the responsiveness to cisplatin chemotherapy. Han demonstrated that p53 regulates the intracellular trafficking of HK2, thereby influencing its localization and function. Phosphorylation of p53 facilitates the recruitment of HK2 and apoptosis-inducing factor (AIF) in chemosensitive ovarian cancer cells, leading to their translocation from mitochondria to the nucleus. This process inhibits HK2-mediated glucose metabolism and enhances cancer cell sensitization for apoptosis induction. Conversely, in p53-mutated resistant cells, mitochondrial HK2 and AIF interact to impede cancer cell apoptosis, highlighting the close association between nuclear HK2 and p53 with chemotherapy sensitivity and improved prognosis 85.

#### LDHA or ALDH

CSC components play a pivotal role in the recurrence of ovarian cancer, with lactic dehydrogenase A (LDHA) or aldehyde dehydrogenase (ALDH) emerging as prominent research areas as independent markers for ovarian CSCs. Casagrande demonstrated that platelet activation leads to the release of cytokines, which may contribute to the expansion of ALDH+/CD133+ ovarian CSCs and diminish patients' sensitivity to chemotherapy drugs like paclitaxel, cisplatin and carboplatin, thereby causing chemotherapy resistance and tumor recurrence 86. Data indicates that SATB1 upregulates ALDH expression and promotes malignant potential by reprogramming energy metabolism in cancer cells. Furthermore, LDHA levels are positively correlated with tumor stage, and combined screening of serum CA-125 and LDHA may enhance the accuracy of prognosis prediction, providing a theoretical foundation for exploring novel anticancer therapies targeting energy metabolism pathways in OC 87.

In summary, dehydrogenases play a pivotal role in the development and progression of ovarian cancer, prompting anti-cancer studies targeting this enzyme. Nowacka observed that ALDH1A1 expression is most abundant in cell lines resistant to paclitaxel or topotecan, which exhibit an upregulation of drug transporters such as P-gp and BCRP. All-trans retinoic acid can deactivate ALDH1A1-mediated NRF2 signaling, thereby inhibiting components of cancer stem cells 88, 89. Xiang also discovered that LDHA positivity could diminish the therapeutic efficacy of Olaparib in certain subtypes of ovarian cancer, suggesting LDHA may serve as a molecular target for combating resistance 90. Developing specific inhibitors against LDHA could potentially enhance the effectiveness of chemotherapy.

#### PDK

Silencing PDK1 or MICU1 leads to mitochondrial Ca2+ overload, stimulating pyruvate dehydrogenase dephosphorylation in OC. This subsequently enhances energy metabolism through the TCA cycle and significantly reduces the proliferation, migration, invasion and matrix degradation abilities of cancer cells 27. Combining PDK1 silencing with cisplatin treatment can decrease mitochondrial transmembrane potential and increase ROS production, thereby enhancing the anti-proliferative and pro-apoptotic activities of cisplatin. The resulting metabolic changes induce cell apoptosis by inhibiting tumor angiogenesis and triggering complex microenvironment reactions as a third effect 91. This study provides new insights into the pathogenesis of OC from the perspective of mitochondrial metabolism and redox homeostasis. Targeting PDK1 may offer a novel therapeutic strategy for normalizing abnormal tumor metabolism.

PDK1 is implicated in tumor immunity. Wang confirmed that elevated levels of PDK1 activate the JNK-c-Jun cascade, leading to increased PD-L1 expression in OC cells. This subsequently inhibits IFN-γ secretion and induces cell apoptosis, impairing the function and survival of CD8+ T cells 92. Additionally, PDK1 can regulate α5β1 integrin to enhance cell adhesion and activate the JNK signaling pathway, resulting in IL-8 expression promotion. Consequently, this promotes cell migration, invasion and angiogenesis 93. The involvement of PDK1 in aerobic glycolysis provides energy for tumor metastasis, highlighting its crucial role in immune response 93. Combining PDK inhibitors with PD-L1 antibodies synergistically enhances antitumor functionality 92, 93.

Liu confirmed that lncRNA AFAP1-AS1 competitively binds to miR-107, leading to the up-regulation of PDK4 levels and subsequently promoting proliferation, migration and invasion in ovarian cancer cells. Additionally, AFAP1-AS1 activates stemness characteristics in subsets of cancer cells to confer resistance against chemotherapy-induced apoptosis 77. Jiang demonstrated that the ALDH+/CD44+ subpopulation derived from ascites also regulates chemotherapy resistance and CSCs characteristics through PDK4 activation of the STAT3/AKT/NF-κB/IL-8 signaling pathway. Furthermore, this subpopulation exhibits invasiveness, clonality, sphere formation ability as well as increased glycolytic flow and decreased mitochondrial oxidative activity which initiate tumor metastasis 94. These findings enhance our understanding of OC pathogenesis and facilitate the identification of potential functional targets.

#### PKM

Studies have demonstrated that PSMD14 deubiquitinates PKM2, leading to an increase in its dimer ratio and nuclear translocation. AXL, Akt2 and follicle-stimulating hormone (FSH) directly target the expression of PKM2, thereby promoting aerobic glycolysis to stimulate proliferation, invasion, migration and cisplatin resistance in ovarian cancer cells 68, 95-97. Simultaneously, overexpression of PKM2 also modulates the expression of multiple tumor-related genes such as upregulating CCND1 while downregulating CDKN1A. This alteration induces proliferation and survival by increasing S phase cells and provides novel insights into comprehending the role of PKM2 in the pathogenic mechanism underlying ovarian cancer 98.

Li confirmed that nitric oxide (NO) plays a dual role in the progression of OC. Mild inflammation stimulates inducible nitric oxide synthase (iNOS) to produce physiological levels of NO, promoting the glycolysis process that induces proliferation of cancer cells and enhances resistance to oxidative stress 99. However, severe inflammation leads to excessive production of NO (≥500 nM), resulting in ROS-induced oxidative damage, inhibition of glycolysis and impairment of cancer cell survival. Further studies have demonstrated that NO induces nuclear translocation of PKM2 through EGFR/ERK2 signaling pathway, thereby positively regulating glycolysis 99. Gao discovered that nitric oxide synthase stabilizes the tetramer structure of PFKM through S-nitrosylation, promoting cancer cell proliferation and tumor growth by inducing glycolysis while reducing macrophage infiltration in the tumor microenvironment 100. Clarifying the molecular mechanisms underlying energy metabolism and immune response induced by nitrogen oxides would greatly benefit patients with OC.

Both the PKM2 inhibitor Compound 3K and the SIRT inhibitor MHY2245 can effectively suppress PKM2 expression and disrupt glycolysis metabolism by modulating the Akt/AMPK/mTOR pathway, thereby inducing G2/M cell cycle arrest, autophagic cell death, and inhibiting cancer cell proliferation as well as colony formation ability 101, 102. This study highlights a promising therapeutic approach targeting the glycolytic pathway in OC. Zhou et al. demonstrated that shikonin downregulates PKM2 expression and triggers DNA oxidative damage and apoptosis through ATM/γH2AX activation 103. Furthermore, it was hypothesized that combining shikonin with Olaparib could inhibit homologous recombination repair, significantly impeding DNA recombination repair in cancer cells 103. These findings provide a feasible strategy to enhance the efficacy of PARP inhibitor maintenance therapy in advanced patients.

#### Other glycolytic enzymes

Phosphoglycerate kinase 1 (PGK1) serves as the initial enzyme in the glycolytic pathway and is responsible for ATP production. Zhang's research revealed that FSH activates the PI3K/Akt pathway, leading to increased expression of ACTL6A, which subsequently upregulates PGK1 and enhances glycolysis levels. This regulation plays a crucial role in tumor growth, proliferation, and EMT 104. These alterations promote invasion ability and offer a potential molecular target for anti-glycolysis therapy in OC. Apart from pivotal glycolytic enzymes, tricarboxylic acid cycle regulators also impact ovarian cancer progression. Isocitrate dehydrogenase 1 (IDH1) catalyzes reversible oxidative decarboxylation from isocitrate to α-ketoglutarate and NADPH. Dahl demonstrated that knocking down IDH1 can enhance histone methylation of E2F target genes while inhibiting their expression, thereby inducing senescence in cancer cells 105. Consequently, this study impedes cell cycle progression and proliferation while providing a novel pro-aging therapy capable of altering metabolism and epigenetics specifically for OC populations.

### GLUT in OC

Since tumor metabolism has gradually emerged as a prominent research focus in anticancer therapy, it has been observed that GLUT1 exhibits abnormal expression across various tumor types. In OC, MUC16 and miR-1204 have been found to positively regulate the expression of GLUT1, thereby facilitating enhanced glucose metabolism reprogramming and glycogen synthesis for increased energy production during tumor progression 82, 106. This study suggests that GLUT1, serving as an energy indicator, can be utilized to assess the prognostic value for OC patients. Notably, targeting GLUTs has yielded significant advancements in this field. For instance, Ma demonstrated the efficacy of BAY-876, a highly selective inhibitor of GLUT1, could suppress cancer cell basal glycolysis and effectively impede tumor growth both in vitro and in vivo. Consequently, BAY-876 holds promising potential as a novel anticancer drug 107.

### Regulatory molecules of aerobic glycolysis in OC

#### HIF-1α

Carcinogenic factors exert resistance to HIF-1α degradation through diverse mechanisms, thereby promoting HIF-1α expression and activating the Warburg effect to regulate the occurrence and development of OC. For instance, GHET1 interacts with VHL to impede VHL-mediated degradation of HIF-1α and induce its protein expression. The upregulation of HIF-1α enhances glucose uptake and lactate production, consequently accelerating malignant progression such as growth, proliferation and metastasis in OC 108. SIK2 not only activates the PI3K/Akt signaling pathway to upregulate HIF-1α expression and induce transcription of glycolytic proteins that facilitate glycolysis but also phosphorylates Drp1 to promote mitochondrial fission. This further inhibits oxidative phosphorylation, inducing cell proliferation, EMT and resistance to apoptosis-all playing pivotal roles in the malignant progression of OC 11.

#### Non-coding RNAs

MiRNAs play a crucial role in regulating the Warburg effect in OC (Table 1). Analysis of miRNA profiles from bone marrow mesenchymal stem cells (BM-MSCs) revealed a negative correlation between miR-1180 levels and the survival of OC patients. Additionally, drug resistance recurrence occurs at a high rate of 93%, which is considered a risk factor for poor prognosis 109. Gu et al. discovered that BM-MSC-released miR-1180 activates Wnt signaling by targeting SFRP1, thereby enhancing glycolysis levels to induce cell proliferation and cisplatin resistance in cancer cells 109. Increasing evidence suggests that lncRNAs, as upstream regulatory molecules of miRNAs, regulate the Warburg effect by sequestering miRNAs in various malignancies 109. For instance, lncRNA LINC00504 downregulates miR-1244 expression and promotes aerobic glycolysis by upregulating glycolysis-related genes in OC cells, leading to increased cell proliferation and apoptosis resistance 75.

CircRNAs also interact with miRNAs to regulate tumor metabolism. The expression of circ-ITCH is decreased in OC. Liu demonstrated that the overexpression of circ-ITCH negatively regulates miR-106a, which subsequently targets the reduction of glycolytic flux to inhibit cancer cell proliferation and invasion while promoting apoptosis 79. In OC tissues, circ-PGAM1, acting as a circular form of phosphoglycerate mutase, is up-regulated. Zhang discovered that there is a negative feedback loop between circ-PGAM1 and miR-542-3p, and down-regulation of miR-542-3p directly targets the expression of CDC5L 80. CDC5L protein binds to the PEAK1 promoter to enhance its transcription, leading to activation of ERK1/2 and JAK2 signaling pathways that promote malignant invasion behavior in OC 80. Therefore, miRNAs may serve as key therapeutic targets and prognostic indicators for improving outcomes (Supplementary Figure 3).

### Signal pathway of aerobic glycolysis in OC

#### Akt signaling pathway

**Figure 2:**
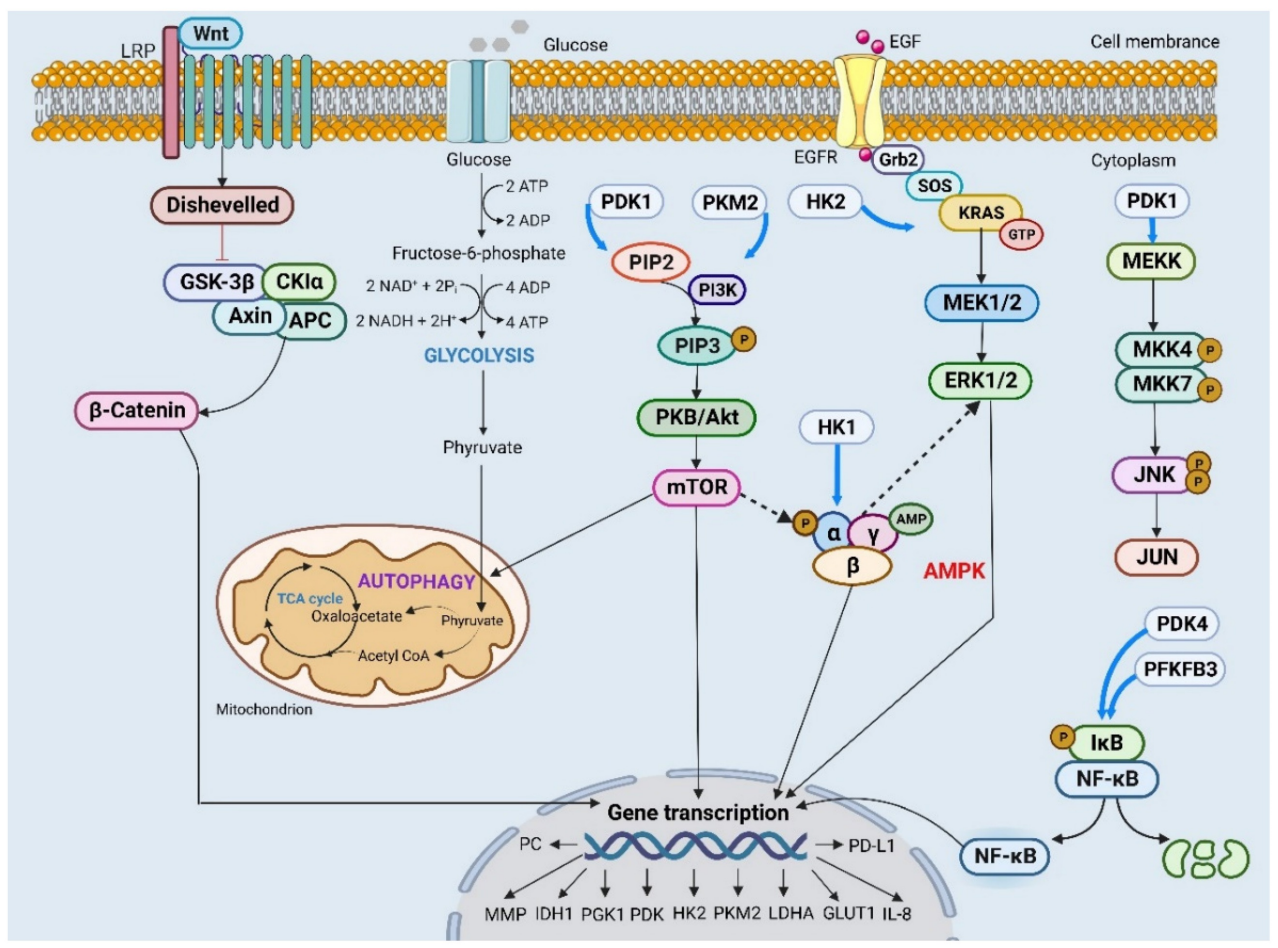
Different signaling pathways affect the expression of key regulatory genes through different mechanisms and then participate in malignant behaviors such as proliferation, invasion and migration in ovarian cancer cells.

Constitutive activation of the PI3K/Akt pathway is a hallmark feature observed in numerous tumor types. For instance, FSH triggers the activation of PI3K/Akt signaling, resulting in the upregulation of ACTL6A and PGK1 expression and induction of glycolytic activity to regulate malignant phenotypes such as cell growth, colony formation and cell viability in OC 104. STAT3 activates Akt and p65, which subsequently release IL-8 through NF-κΒ signaling to modulate the characteristics of ovarian cancer stem cells 94. MHY2245, LY294002 and Compound 3K specifically target key enzymatic activities involved in glycolysis regulation including glucose uptake, pyruvate conversion and lactate production 97, 101, 102. Moreover, these compounds also inhibit cell growth by inducing autophagy and apoptosis mediated by the Akt/mTOR signaling pathway in OC. Therefore, it is plausible to develop anticancer drugs based on targeting the Akt signal (Figure 2).

#### Wnt signaling pathway

The Wnt signaling pathway plays a crucial role in regulating ovarian cancer progression. Specifically, BM-MSCs release miR-1180 to activate the Wnt signaling pathway in cancer cells, thereby upregulating glycolysis levels and enhancing chemotherapy resistance. This confirms that the Wnt signaling pathway is a key factor contributing to drug resistance and recurrence in OC patients 109. Additionally, Yang demonstrated that TNKS not only activates the Wnt/β-catenin signaling pathway to increase pyruvate carboxylase levels and promote malignant potential induced by aerobic glycolysis such as proliferation, migration, and invasion of cancer cells but also regulates cell cycle progression and resists apoptosis to reduce chemotherapy sensitivity. In contrast, XAV939 inhibits the Wnt/β-catenin pathway and reduces expression of Cyclin D, MMP-9, snail and MDR proteins which may lead to more reliable treatment outcomes for OC patients 110.

#### MAPK signaling pathway

MAPK is a crucial signal transduction pathway in OC. HK2 acts as a mediator to facilitate glucose metabolic reprogramming, thereby enhancing the malignant potential and promoting cell autophagy through FAK/MAPK kinase signaling to confer resistance against cisplatin therapy in ovarian cancer stem cells 13. Simultaneously, activation of the downstream MMP9/NANOG/SOX9 cascade promotes EMT and stem cell characteristics 13. Li discovered that HK1 also upregulates glycolytic flux via MAPK/ERK signaling, consequently promoting the proliferation, invasion and migration processes of OC cells. This suggests that targeting HKs and AMPK oncogenic signals may hold promise for developing novel anticancer drugs targeting the aerobic glycolysis pathway 14.

### Immunity and aerobic glycolysis in OC

Glycolysis metabolism and the tumor microenvironment, driven by aerobic glycolysis, contribute to immune evasion. The investigation of immune characteristics associated with tumor-infiltrating lymphocytes may offer a scientific foundation and clinical value for immunotherapy. PDK1 regulates PD-L1 expression through the JNK-c-Jun pathway, thereby inhibiting infiltration of CD8+ T cells and promoting the formation of an immunosuppressive microenvironment in OC 92. Gao demonstrated that PGK1 induces the expression of the premetastatic chemokine CXCL8, which recruits neutrophils to facilitate EMT processes and immune escape 111. IL-8 is a highly expressed angiogenic chemotactic factor that binds to endothelial cell surface receptors CXCRs, mediating tumor angiogenesis effects in both primary ovarian cancer and ascitic metastatic carcinomas. Meanwhile, PDK4 regulates IL-8 to modulate properties of CSCs, both of which can induce the acquisition of a metastatic phenotype by cancer cells 93, 94. These studies aid in comprehending the intricate regulatory mechanisms between glycolytic genes and tumor immune evasion, while providing preclinical evidence for developing anti-tumor immunotherapy.

### Therapeutic value of aerobic glycolysis in OC

Studies have demonstrated that metformin exerts its regulatory effects on ovarian cancer glucose metabolism by targeting mitochondrial respiratory chain complex I, thereby restoring chemotherapy sensitivity. For instance, metformin not only downregulates the expression of PDK1 to overcome cisplatin resistance but also reduces the levels of HIF-1α in drug-resistant cancer cells and interferes with the transcription of glucose metabolism-related genes. These actions lead to a decrease in ATP content, impair cell survival and restore cisplatin activity, ultimately improving treatment efficacy 112, 113. These findings support the notion that combining metformin with first-line chemotherapy is an effective strategy for patients with resistant or metastatic symptoms aiming to enhance chemotherapy resistance. However, it should be noted that high glucose levels can induce oxidative stress response and lower glycoside hydrolase activities as well as HIF-1α levels in diabetic OC patients. Consequently, this hinders the toxic effects of metformin on cancer cell growth and proliferation 114. Therefore, it is imperative to identify patient subgroups who may benefit from metformin treatment in order to develop more precise clinical strategies 114.

Berberine and ginsenoside 20(S)-Rg3 are commonly considered as adjunctive medications for the treatment of malignant tumors. They counteract DNMT3A-mediated methylation in the promoter region of various miRNA precursor genes, which subsequently negatively regulates the Warburg effect and impedes malignant behaviors in cancer cells 73, 115. Zhang confirmed that 20(S)-Rg3 also modulates lncRNA expression profiles, such as H19 upregulating the levels of miR-324-5p and miR-603, thereby blocking ovarian cancer progression through inhibition of the Warburg effect 73, 78. Heme serves as a crucial cofactor for mitochondrial oxidative phosphorylation in electron transport and ATP synthesis. Kaur discovered that exogenous supplementation of the heme precursor 5-aminolevulinic acid enhances heme synthesis to disrupt Bach1 stability, activate AMPK and induce antioxidant responses, consequently inhibiting glycolysis, mitochondrial respiration and cell proliferation 116. These studies elucidate the potential application of metabolic interference in cancer therapy while providing reliable evidence for screening safe and effective clinical drugs.

In addition to the aforementioned anti-cancer drug, researchers have also enhanced the activity of anticancer drugs by modifying their delivery modes. Zhang designed HK2 shRNA nanoparticles that were modified with FSH peptide as a carrier, taking advantage of its specific expression in ovarian tissue. This targeted delivery system enables efficient drug absorption and reduces side effects, resulting in selective accumulation and potent anti-tumor effects 69. These findings confirm the feasibility of RNAi-based drugs and nanodrug delivery systems. Shen explored intraperitoneal radiotherapy systems by injecting radionuclide anticancer agent 188Re-liposome into the abdominal cavity, which alters energy metabolism through p53 activation and effectively eliminates ovarian CSC-like cells 117.

In OC, it has been further confirmed that key proteins such as HK2, LDH, PDK, PKM2, GLUT, and transcription factors like HIF-1 and non-coding RNAs regulate malignant progression through diverse mechanisms and signaling pathways. Similarly, the close association between glycolytic metabolism and immune escape of cancer cells has been established, validating the feasibility of designing immunotargeted drugs. Moreover, apart from the anticancer effects of metformin, berberine and ginsenoside; a significant finding is that researchers have achieved drug-specific blocking effects by altering the mode of administration. Whether utilizing carriers for HK2 shRNA nanoparticles or employing intraperitoneal injection of radioactive anti-cancer agents, both approaches enhance therapeutic efficacy and improve patient survival rates. The application of these novel technologies holds promising prospects for ovarian cancer patients in the near future.

## Conclusions

Metabolic syndrome encompasses a range of pathological conditions characterized by disruptions in glucose, lipid and protein metabolism, including diabetes, obesity, hypertension, cardiovascular diseases, cerebrovascular diseases, etc. Gynecological tumors can be considered as metabolic disorders. The oxidative stress and systemic inflammation induced by metabolic syndrome have the potential to activate the immune system in tumor patients. In this inflammatory milieu, tumor cells reprogram their metabolic pathways to sustain high proliferation rates and facilitate tumor growth, invasion and neovascularization while evading cell death signals. Consequently, these alterations ultimately contribute to unfavorable patient outcomes.

In this article, we provide a comprehensive overview of the carcinogenic role of aerobic glycolysis and explore the potential therapeutic value of targeting glucose metabolism in three major gynecological tumors. Our aim is to elucidate the underlying molecular mechanisms and pathological regulatory networks involved in tumor occurrence and development. Additionally, we highlight recent advancements in the treatment of glucose metabolism and identify several commonly used clinical drugs with proven anti-tumor activity, including metformin, berberine and ginsenoside. Notably, metformin exhibits promising therapeutic potential across all three tumor types. Furthermore, our analysis reveals both similarities and differences in glucose metabolism among different tumors. These findings have significant implications for developing personalized treatment strategies for cancer patients. However, it is important to acknowledge that there are still limitations within current research efforts. For instance, many targeted molecular inhibitors remain at the pre-clinical stage and require further evaluation before being incorporated into routine diagnosis and treatment protocols. Therefore, multi-center large-scale trials are necessary to assess their clinical application value in the future. Moreover, while most research data originate from cell experiments, it is crucial to consider that human energy metabolism is highly complex. Consequently, additional investigations are warranted to analyze how glucose metabolism interacts with lipid metabolism and protein metabolism in influencing the biological behavior of gynecologic oncology patients. By gaining a deeper understanding of these interactions through metabolic reprogramming studies, novel anti-tumor strategies can be developed.

Obesity, being the primary determinant of metabolic syndrome, can facilitate the development of insulin resistance, hyperinsulinemia and hyperglycemia. Huang's review highlights the reprogramming of glucose metabolism in EC. Evidence suggests that adipokines related to obesity, along with insulin resistance and metabolic alterations, serve as pivotal carcinogenic factors that render therapeutic strategies targeting cellular metabolic processes feasible. Effectively managing metabolic syndrome through weight loss and reducing lipid and glucose levels represents an efficacious approach for treating and preventing EC 118. Our review also indicates a significantly higher likelihood of EC among patients with diabetes, obesity or hyperlipidemia compared to normal women. This mechanism may involve energy metabolic reprogramming which aligns with Huang's findings. Furthermore, we discovered that insulin or IGF-1 binds to specific receptors on tumor cell membranes to regulate tumor growth and glucose metabolism by activating the PI3K/AKT/mTOR pathway in EC, meanwhile metformin can reduce plasma IGF-1 concentration thereby exerting anticancer activity 119. Su conducted a review on HIF-1α/ERR α-mediated metabolic reprogramming in EC, elucidating the various signaling pathways through which HIF-1α and ERRα regulate energy metabolism to confer resistance to heat-induced apoptosis in tumor cells 120. The study also explored the regulatory role of HIF-1α/ERRα in preventing and treating EC, providing new insights into the molecular mechanisms underlying cancer progression. Additionally, we summarized the molecular mechanism by which HIF-1α regulates glycolysis in EC and speculated that HIF-1α/ERRα may mediate pathological processes by regulating glycolytic enzyme spectrum signaling pathways. Apart from EC, HIF-1α also plays a regulatory role in energy metabolism of CC and OC 9, 108. We confirmed that hypoxia induces high expression of highly active aerobic glycolysis via upregulation of HIF-1α, promoting tumor progression by providing energy and macromolecular substances. Hyperglycemia can also induce GLUT4 expression through ERβ signals 61, leading to increased glucose intake that provides more energy for EC growth and ultimately accelerates disease progression 61.

Kobayashi concluded that the metabolic profiles of ovarian CSCs and non-CSCs are influenced by various factors in the tumor microenvironment, such as normal oxygen levels and hypoxia, quiescence and proliferation states, as well as nutrient supply and starvation conditions. Metabolic stress, including genomic and epigenetic changes in metabolic-related genes, may regulate energy homeostasis in both cancer cells and host cells 121. Additionally, we found that ovarian CSCs exhibit a more aggressive phenotype requiring ample energy supply to meet their rapid growth and proliferation demands. ALDH serves as a unique and sensitive marker for identifying ovarian CSCs. It has been confirmed that ovarian cancer cells can adapt to environmental challenges by switching between glycolysis and oxidative phosphorylation pathways. Chen reviewed the latest research progress on miRNAs in gynecological malignancies. MiRNAs with significantly dysregulated expression in serum samples or tissues, along with their target genes associated with tumors, may play crucial roles in tumor diagnosis and serve as potential therapeutic options 12. We also summarized the miRNAs involved in regulating glucose metabolism in gynecologic tumors (Table 1). These miRNAs participate in multiple aspects of tumor development, including occurrence, proliferation, invasion, chemoradiotherapy resistance and immune evasion. Meanwhile, we also highlight their importance as key targets for diagnosis and treatment of gynecologic tumors. We have also observed a close association between lncRNAs and circ-RNAs with miRNAs, and their combined interactions play a significant role in the initiation and progression of tumors. Hu's study concluded that SIK2 not only enhances the Warburg effect by promoting glycolysis, inhibiting oxidative phosphorylation and gluconeogenesis, but also regulates intracellular lipid metabolism by promoting lipid synthesis and FAO. Ultimately, this leads to growth, proliferation, invasion, metastasis and therapeutic resistance in OC 122. Additionally, molecules such as HSP70, SATB1, PSMD14, and STAT3 regulate glucose metabolism through different mechanisms or signaling cascades in OC, which promotes cancer progression and results in poor prognosis 84, 87, 94, 95. These targets could potentially serve as key focal points for future exploration of targeted drugs for OC.

The aforementioned studies have investigated the correlation between gynecologic tumors and metabolism from various perspectives, encompassing glucose metabolism, lipid metabolism and protein metabolism. However, they lacked a comprehensive and systematic overview of the relationship between common gynecologic tumors and key targets within aerobic glycolysis signaling pathways. From a novel standpoint, we will concentrate on elucidating the role and mechanism of proteins related to aerobic glycolysis, as well as key regulatory molecules and signaling pathways in cervical cancer, endometrial cancer and ovarian cancer in the review. Additionally, we aim to propose fresh insights into tumor metabolism therapy with the intention of establishing a connection between gynecological tumors and energy metabolism. This will provide researchers with a foundation for translating basic research into clinical applications by designing clinical trials based on the metabolic characteristics of gynecological tumors. Furthermore, it will facilitate the study of new drugs for targeted single-agent therapy or their combination with existing cytotoxic drugs in order to offer scientific support for future endeavors.

To summarize, due to the highly flexible metabolic pathways in tumors, not only glucose metabolism but also lipid metabolism and amino acid metabolism are reprogrammed in tumor cells. The intricate network of metabolic interactions between tumor cells and the surrounding microenvironment creates unique metabolic conditions that collectively contribute to tumor initiation and malignant progression. Although aerobic glycolysis-associated metabolic pathways have been implicated in the development and progression of gynecological malignancies, research on the interplay between cellular glycolipid metabolism and immune response to these metabolic alterations is still lacking. Understanding the precise metabolic dependency, compensatory mechanisms and immune microenvironment's response to the metabolic state may offer a novel target for treating gynecological tumors. Given the existing treatments, further research is needed to stratify tumor patients based on different metabolic states and determine personalized treatment regimens. Tumor metabolism research has entered an exciting era where innovative strategies targeting specific metabolic profiles will become more applicable.

## Supplementary Material

Supplementary figures.

## Figures and Tables

**Table 1:** miRNAs and their targets in aerobic glycolysis in three major gynecological tumors

Glycolytic protein	MiRNAs	Role	Expression	Mechanism	Type of cancer		References
HK2	miR-145, miR-148a, miR-497	CSCs, chemotherapy resistance	down	/		CC	18
	miR-9-5p	Tumor suppressor	down	AKT/mTOR, p53		CC	17
	miR-143-3p	-	down	Myc		CC	70
	circCDKN2B-AS1	Tumor suppressor	up	IMP3		CC	71
LDHA	miR-34a	chemotherapy sensitivity	down	lncRNA NEAT1		CC	23
PDK1	miR-126-3p	Tumor suppressor	down	Bcl-xL, Bax, caspase 3/7		CC	28
	miR-155-5p	autophagy	down	LC3, P62		CC	29
PDK4	miR‐103a‐3p	Tumor promoter	down	ncRNA LINC00662		CC	30
	miR-16-5p	chemotherapy resistance	down	/		CC	31
PKM2	miR-let-7a	Tumor suppressor	down	/		CC	72
IDH2	miR-124-5p	Tumor promoter	down	HIF-1α		CC	44
HK2	miR-455, miR-181a	EMT	down	DLEU2, EZH2		EC	55
HK2	miR-603	Tumor suppressor	down	20(S)-Rg3, DNMT3A		OC	73
	miR‐145	/	down	DNMT3A		OC	74
	miR-1244	Tumor promoter	down	lncRNA LINC00504		OC	75
LDHA	miR-383	Tumor suppressor	down	/		OC	76
PDK4	miR-107	Tumor promoter	down	lncRNA AFAP1-AS1		OC	77
PKM2	miR-324-5p	Tumor suppressor	down	20(S)-Rg3, H19		OC	78
	miR-1244	Tumor promoter	down	lncRNA LINC00504		OC	75
PDK1	miR-1244	Tumor promoter	down	lncRNA LINC00504		OC	75
CDH1	miR-106a	Tumor promoter	down	circ-ITCH		OC	79
circ-PGAM1	miR-542-3p	Tumor promoter	down	CDC5L, PEAK1		OC	80
GADPH	miR-125b	Tumor promoter	down	STAT3		OC	81
GLUT-1	miR-1204	Tumor promoter	up	/		OC	82
							

## Abbreviations

CC: Cervical cancer
EC: Endometrial carcinoma
OC: Ovarian cancer
HR-HPV: High-risk human papillomavirus infection
EMT: Epithelial-mesenchymal transformation
HCC: Hepatocellular carcinoma
GLUT: Glucose transporters
ATP: Adenosine triphosphate
ROS: Reactive oxygen species
MMPs: Matrix metalloproteinases
HK2: Hexokinase 2
G-6-P: Glucose-6-phosphate
CSCs: Cancer stem cells
lncRNA: Long non-coding RNA
LDHA: Lactic dehydrogenase A
OS: overall survival
PFS: progression free survival
PDK: Pyruvate dehydrogenase kinase
TCA cycle: tricarboxylic acid cycle
PKM2: Pyruvate kinase M2
ALDOA: aldolase A
ENO1: enolase 1
HIF-1α: hypoxia inducible factor-1α
miRNAs: MicroRNAs
FAK: focal adhesion kinase
ALDH: Aldehyde dehydrogenase
PFKFB3: PFK158, a Fructose 2,6-Bisphosphatase 3
PGI: Phosphoglucose isomerase
AMF: autocrine motility factor
SGLT1: Sodium/Glucose Cotransporter 1
AIF: apoptosis-inducing factor
FSH: follicle-stimulating hormone
NO: nitric oxide
PGK1: Phosphoglycerate kinase1
IDH1: Isocitrate dehydrogenase 1
BM-MSCs: bone marrow mesenchymal stem cells
