# Supplementary material for: Glucose metabolism reprogramming in gynecologic malignant tumors
Source: J Cancer. 2024 Mar 17;15(9):2627–45. doi: 10.7150/jca.91131 (PMC10988310; doi:10.7150/jca.91131)

Supplementary Figure 1. These molecules, such as p53, HIF-1α, miRNAs and c-Myc, may play a vital role in regulating aerobic glycolysis of cervical cancer through different signals and mechanisms.

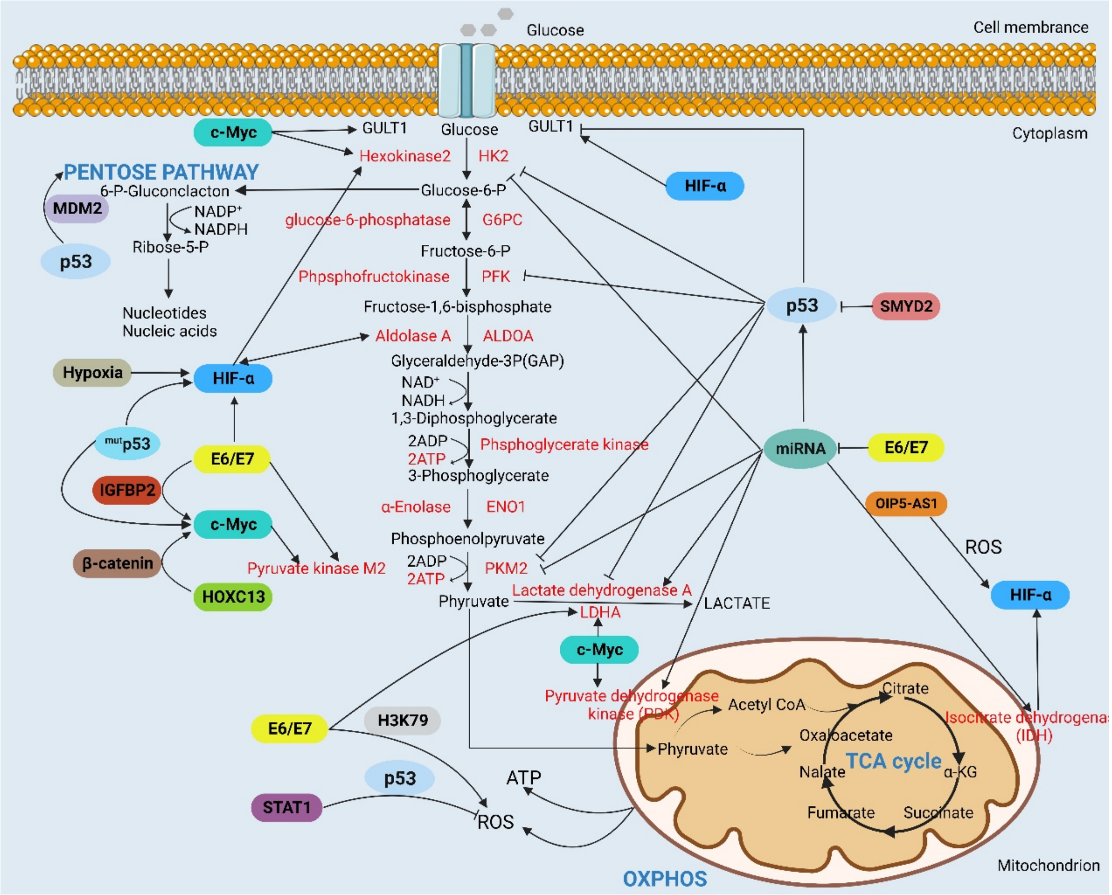

Supplementary Figure 2. These may play a vital role in regulating aerobic glycolysis of endometrial cancer through different signal molecules and mechanisms.

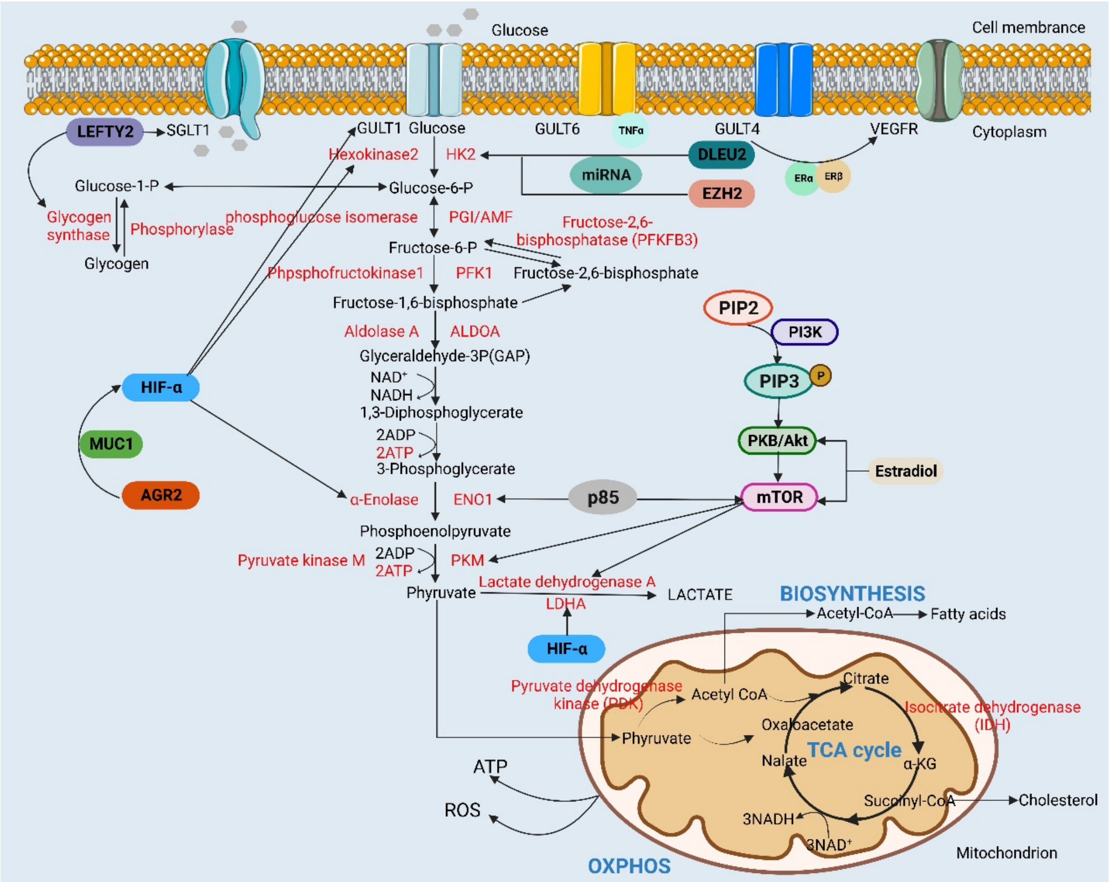

Supplementary Figure 3. These molecules, such as p53, HIF-1α and non-coding RNAs, may play a vital role in regulating aerobic glycolysis of ovarian cancer through different signals and mechanisms.

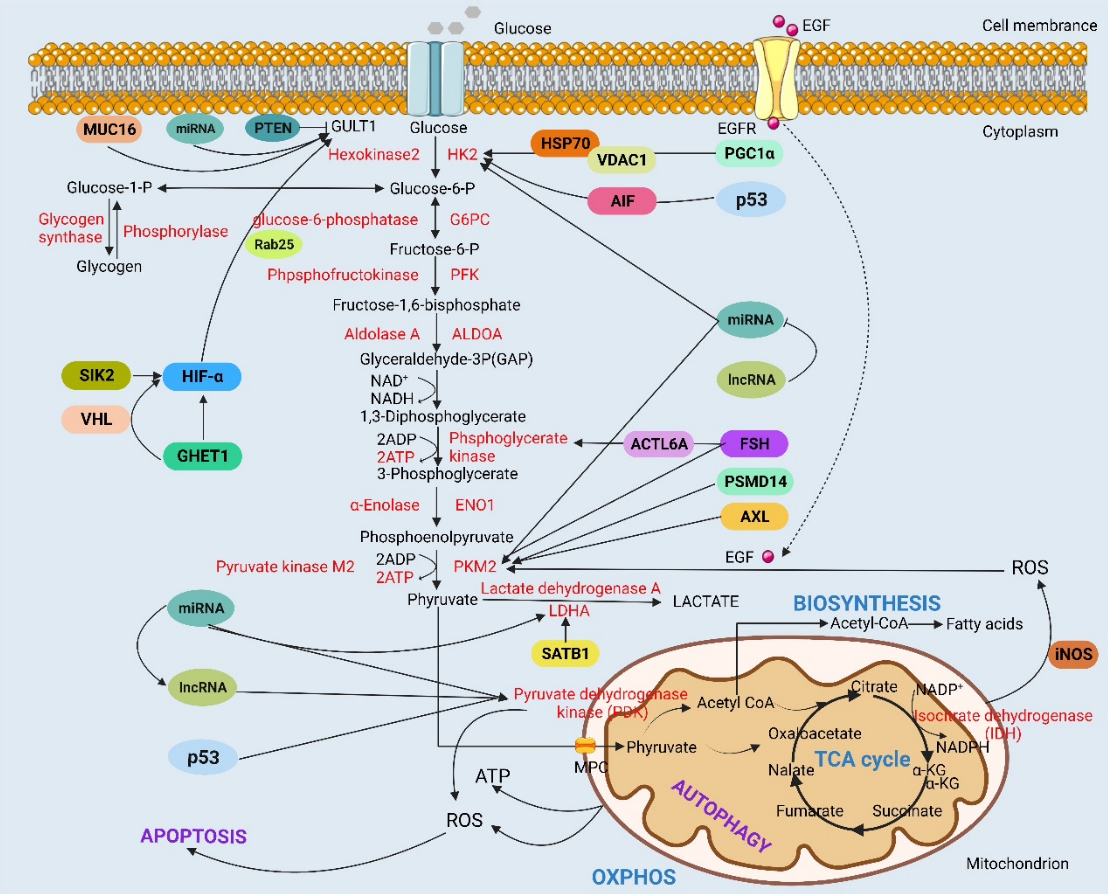

Supplement: Supplementary file 1 — Supplementary figures. [file jcav15p2627s1.pdf]
